# Performance evaluation of SimPET-L and SimPET-XL: MRI-compatible small-animal PET systems with rat-body imaging capability

**DOI:** 10.1186/s40658-023-00534-x

**Published:** 2023-03-07

**Authors:** Minjee Seo, Guen Bae Ko, Kyeong Yun Kim, Jeong-Whan Son, Jung Woo Byun, Yun-Sang Lee, Kyeong Min Kim, Jang Woo Park, Kipom Kim, Taekwan Lee, Jae Sung Lee

**Affiliations:** 1 Department of Biomedical Sciences, Seoul National University College of Medicine, Seoul, 03080 South Korea; 2 Department of Nuclear Medicine, Seoul National University College of Medicine, 103 Daehak-ro, Jongno-gu, Seoul, 03080 South Korea; 3 Brightonix Imaging Inc., Seoul, 04782 South Korea; 4 Korea Institute of Radiological and Medical Sciences, Seoul, 01812 South Korea; 5 Brain Research Core Facility, Korea Brain Research Institute, 61 Cheomdan-ro, Dong-gu, Daegu, 41062 South Korea

**Keywords:** PET/MRI, Molecular imaging, NEMA performance, Instrumentation

## Abstract

**Background:**

SimPET-L and SimPET-XL have recently been introduced with increased transaxial fields of view (FOV) compared with their predecessors (SimPET™ and SimPET-X), enabling whole-body positron emission tomography (PET) imaging of rats. We conducted performance evaluations of SimPET-L and SimPET-XL and rat-body imaging with SimPET-XL to demonstrate the benefits of increased axial and transaxial FOVs.

**Procedures:**

The detector blocks in SimPET-L and SimPET-XL consist of two 4 × 4 silicon photomultiplier arrays coupled with 20 × 9 array lutetium oxyorthosilicate crystals. SimPET-L and SimPET-XL have an inner diameter (bore size) of 7.6 cm, and they are composed of 40 and 80 detector blocks yielding axial lengths of 5.5 and 11 cm, respectively. Each system was evaluated according to the National Electrical Manufacturers Association NU4-2008 protocol. Rat imaging studies, such as ^18^F-NaF and ^18^F-FDG PET, were performed using SimPET-XL.

**Results:**

The radial resolutions at the axial center measured using the filtered back projection, 3D ordered-subset expectation maximization (OSEM), and 3D OSEM with point spread functions correction were 1.7, 0.82, and 0.82 mm FWHM in SimPET-L and 1.7, 0.91, and 0.91 mm FWHM in SimPET-XL, respectively. The peak sensitivities of SimPET-L and SimPET-XL were 6.30% and 10.4% for an energy window of 100–900 keV and 4.44% and 7.25% for a window of 250–750 keV, respectively. The peak noise equivalent count rate with an energy window of 250–750 keV was 249 kcps at 44.9 MBq for SimPET-L and 349 kcps at 31.3 MBq for SimPET-XL. In SimPET-L, the uniformity was 4.43%, and the spill-over ratios in air- and water-filled chambers were 5.54% and 4.10%, respectively. In SimPET-XL, the uniformity was 3.89%, and the spill-over ratio in the air- and water-filled chambers were 3.56% and 3.60%. Moreover, SimPET-XL provided high-quality images of rats.

**Conclusion:**

SimPET-L and SimPET-XL show adequate performance compared with other SimPET systems. In addition, their large transaxial and long axial FOVs provide imaging capability for rats with high image quality.

## Background

Small-animal imaging is an important tool in preclinical research. Various high-resolution imaging techniques for small animals are used to understand the pathophysiology of human diseases and investigate the in vivo kinetics and therapeutic efficacy of pharmaceuticals under development [1–3]. In particular, positron emission tomography (PET) is a functional and molecular imaging modality that enables the quantitative evaluation of various biological processes [4]. In addition, small-animal PET scanners with high spatial resolution and sensitivity allow for active translational research using existing and new radiotracers [5–7].

As in clinical studies, hybrid molecular imaging systems in which a PET scanner is combined with computed tomography (CT) or magnetic resonance imaging (MRI) are commonly used in small-animal studies. Although PET/CT is more widely used than PET/MRI, the various advantages of MRI over CT are increasing the use of PET/MRI in small-animal studies [8–12]. Compared with CT, MRI provides better soft-tissue contrast, allowing for a more accurate anatomical localization of the radiotracer distribution. In addition, simultaneous PET/MRI scans can improve the spatiotemporal correlation of functional and anatomical information provided by these two imaging modalities [13–15]. Simultaneous PET/MRI acquisition also reduces the overall scan time and anesthesia usage. The multiparameter information provided by different MRI pulse sequences is another important strength of PET/MRI in small-animal studies [16, 17].

The SimPET series of Brightonix Imaging are advanced silicon photomultiplier (SiPM)-based small-animal PET inserts that allow simultaneous PET/MRI [18, 19]. Recently, SimPET-X was introduced with 2× longer axial field of view (FOV) of 11 cm than the previous SimPET version, enabling total-body imaging of a mouse in a single bed position. For SimPET-X, the analog pulse shape is optimized to handle the large amount of SiPM signals generated owing to the significantly increased sensitivity [19]. In addition, the resolution-recovery reconstruction algorithm accelerated by a graphics processing unit improves the spatial resolution. However, in SimPET-X, owing to the 6 cm inner diameter of the detector ring, size is limited for rat-body imaging when used in standalone mode.

SimPET-L and SimPET-XL are newly developed PET inserts intended for small-animal imaging with an increased transaxial FOV compared with their predecessors (SimPET™, SimPET-S and SimPET-X). The larger inner and outer diameters (7.6 and 11.2 cm, respectively) of SimPET-L and SimPET-XL compared with previous scanners allow for more space for accommodating animals and radiofrequency coils when combined with MRI systems, with gradient coils having inner diameter of 11.2 cm or more (e.g., Bruker BioSpec 70/20 and 94/20).

In this study, we assessed the performance of SimPET-L and SimPET-XL based on the National Electrical Manufacturers Association (NEMA) NU4-2008 protocol and compared the performances across the SimPET series systems. Furthermore, we conducted rat-body imaging studies using SimPET-XL to demonstrate the benefits of increased axial and transaxial FOVs.

## Methods

### SimPET-L and SimPET-XL systems

**Fig. 1:**
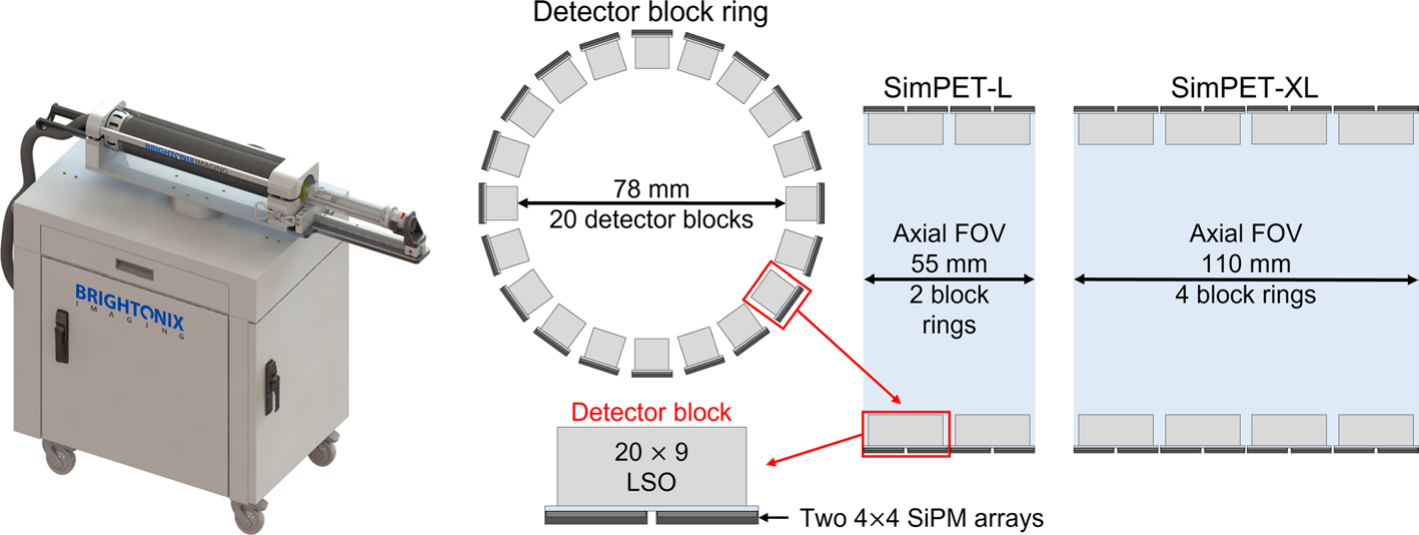
System configuration of SimPET-L and SimPET-XL systems

The detector blocks in SimPET-L and SimPET-XL consist of two 4 × 4 SiPM arrays (S14161-3050HS-04; Hamamatsu Photonics, Japan) coupled with a 20 × 9 array of lutetium oxyorthosilicate (LSO) crystals. The dimension of the photosensitive area of each SiPM pixel is 3 × 3 mm^3^ and the dimension of each crystal element is 1.2 × 1.2 × 10 mm^3^. SimPET-L is composed of 40 detector blocks yielding two-block rings, and SimPET-XL is composed of 80 detector blocks yielding four-block rings. The detailed PET system configurations are depicted in Fig. 1.

### Performance evaluation

In accordance with the NEMA NU4-2008 protocol and measurement methods used in previous studies in [18, 19], SimPET-L and SimPET-XL were evaluated in terms of spatial resolution, sensitivity, count rate, and image quality (IQ).

#### Spatial resolution

A ^22^Na point source (0.14 MBq) embedded in a 10 mm acrylic cube was scanned to measure the spatial resolution. Data were acquired with the source placed at the center and one-fourth of the axial FOV moving radially from the center of the PET detector ring up to 24 mm with a step size of 6 mm. PET data at each position were acquired for 5 min in an energy window of 350–650 keV. The data were reconstructed using filtered back projection (FBP) reconstruction as specified in the NEMA standard. Also, additional reconstructions were performed using a 3D ordered-subset expectation maximization algorithm (OSEM) (12 subsets and 3 iterations) as well as 3D OSEM with point spread functions (PSF) correction (12 Subsets and 3 iterations). The results were reported by calculating the full width at half maximum (FWHM) and full width at tenth maximum (FWTM) according to the NEMA NU4-2008 protocol.

#### Sensitivity

For sensitivity measurements, the same ^22^Na point source used for the spatial resolution measurements was employed. The source was moved along the axis of the systems with a step size of 0.64 mm. Data were acquired for 1 min in various energy windows: 100–900, 250–750, 350–650, and 400–600 keV. The absolute sensitivity was reported according to the NEMA protocol.

#### Count-rate performance

The count rate performance was measured using a NEMA mouse-like phantom (70 and 25 mm in length and diameter, respectively) with an energy window of 250–750 keV. A line source (length of 60 mm) filled with ^18^F solution was inserted into a hole with a diameter of 3.2 mm in the NEMA mouse-like phantom. The scan duration was 10 min, and data were acquired every 10 min over 24 h. The prompt, random, scatter, and true event rates and noise-equivalent count rate (NECR) were reported as described in the NEMA protocol. The scatter fraction was calculated using the data acquired when the random event rate was less than 1.0% of the true event rate.

#### IQ phantom

An IQ phantom compliant with the NEMA NU4-2008 protocol was scanned to estimate the performance of the imaging systems. The scan data were obtained for 20 min with an energy window of 350–650 keV and reconstructed using the 3D ordered-subset expectation maximization algorithm (12 subsets and 4 iterations for SimPET-L, 12 subsets and 6 iterations for SimPET-XL) with attenuation and scatter corrections as well as normalization. The performance of the imaging system was assessed in terms of uniformity, recovery coefficients (RCs), and spill-over ratio (SOR).

### Animal imaging experiments

Rat imaging studies were performed to explore the advantages of the rat-body imaging and high sensitivity of the standalone SimPET-XL configuration. The animal studies were approved by the Institutional Animal Care and Use Committee of Seoul National University Hospital (SNU-220113-7-1). During the imaging studies, rats were placed on an animal-handling system in a prone position while anesthetized with isoflurane (2% in air). A whole-body bone PET scan of a 431.67 g Sprague–Dawley rat (male, 10 weeks old) was performed for 40 min using two-bed positions 60 min after intravenous injection of 36.6 MBq ^18^F-NaF. In addition, other PET scans were performed on a 140.83 g Sprague–Dawley rat (male, 6 weeks old) and a 469.6 g Sprague–Dawley rat (male, 10 weeks old) for 20 min using one bed position 60 and 40 min after intravenous injection of 33.6 and 33.4 MBq ^18^F-FDG, respectively.

## Results

### Performance evaluation

#### Spatial resolution

**Fig. 2:**
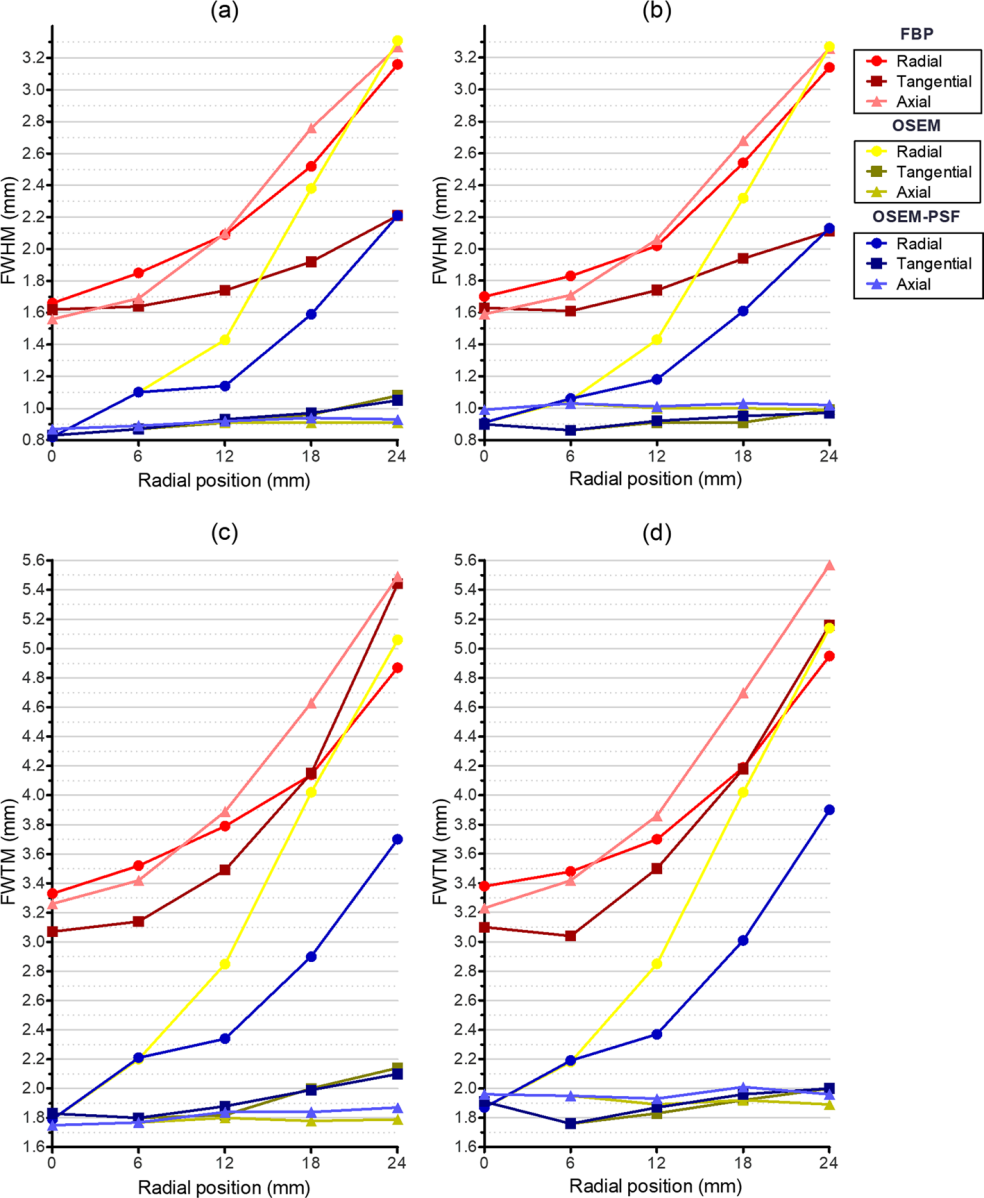
Spatial resolution of **a** SimPET-L (FHWM), **b** SimPET-XL (FWHM), **c** SimPET-L (FWTM), and **d** SimPET-XL (FWTM) measured using FBP, OSEM, and OSEM-PSF at axial center per radial position

The spatial resolutions (axial, radial, and tangential) of SimPET-L and SimPET-XL were obtained according to the radial position, as shown in Fig. 2. Almost of the spatial resolutions measured using the FBP algorithm were worse than the others (OSEM and OSEM-PSF) and above 1.5 mm FWHM. The radial spatial resolution of SimPET-L measured using the FBP, OSEM, and OSEM-PSF were 1.7 mm FWHM (3.3 mm FWTM), 0.82 mm FWHM (1.8 mm FWTM), and 0.82 mm FWHM (1.8 mm FWTM) at the center of the axial center (Fig. 2a). The radial spatial resolution of SimPET-XL measured using the FBP, OSEM, and OSEM-PSF were 1.7 mm FWHM (3.4 mm FWTM), 0.91 mm FWHM (1.9 mm FWTM), and 0.91 mm FWHM (1.9 mm FWTM) at the center of the axial center (Fig. 2b). The radial spatial resolution at the 24 mm off-center position was improved by using OSEM algorithm (2.2 mm FWHM in SimPET-L and 2.1 mm FWHM in SimPET-XL) with PSF corrections compared to FBP algorithm (3.2 mm FWHM in SimPET-L and 3.1 mm FWHM in SimPET-XL) and OSEM (3.3 mm FWHM in SimPET-L and 3.3 mm FWHM in SimPET-XL).

#### Sensitivity

**Fig. 3:**
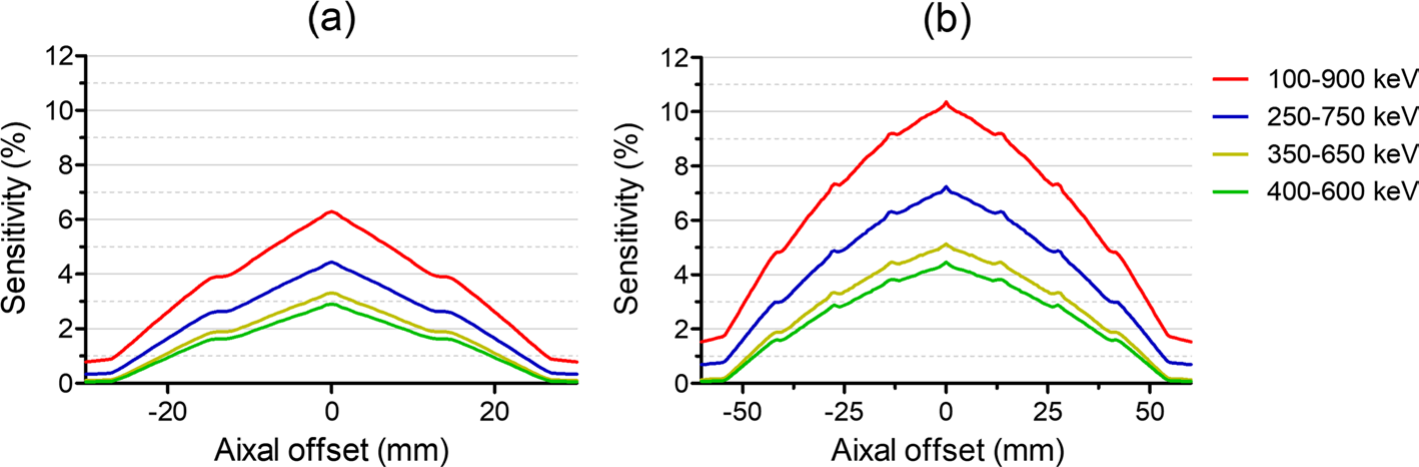
Absolute sensitivity at transaxial center with various energy windows for **a** SimPET-L and **b** SimPET-XL

The absolute peak sensitivities were 6.30% for SimPET-L and 10.4% for SimPET-XL with an energy window of 100–900 keV and 4.44% for SimPET-L and 7.25% for SimPET-XL with an energy window of 250–750 keV. Figure 3 shows the axial profile of the sensitivity for each system. The absolute peak sensitivities of different energy windows are listed in Table 2.

#### Count-rate performance

**Fig. 4:**
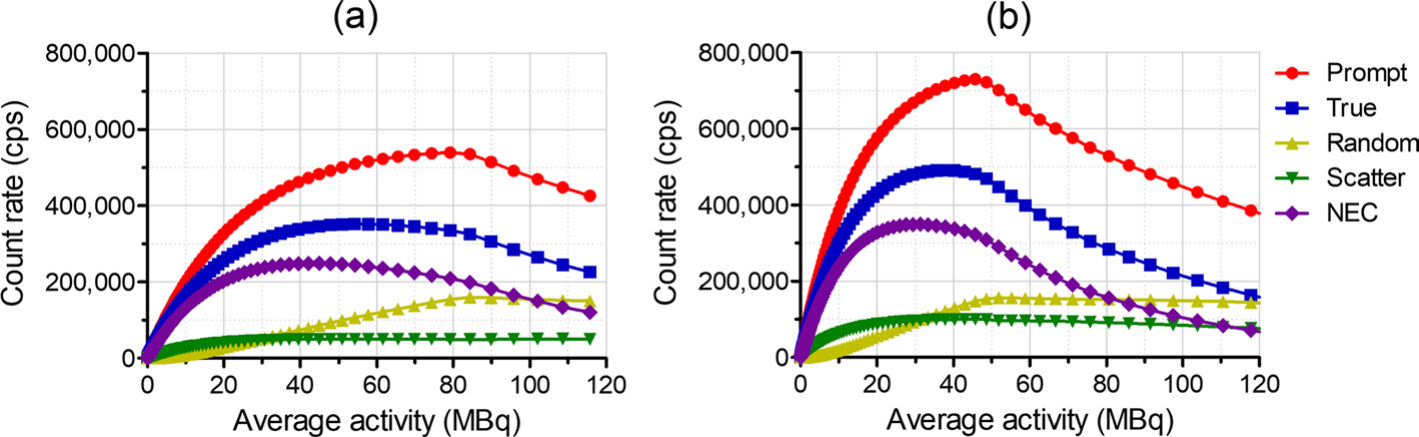
Count-rate performance with energy window of 250–750 keV for **a** SimPET-XL and **b** SimPET-L

SimPET-L yielded a peak NECR of 249 kcps at 44.9 MBq and scatter fraction of 17.4% for an energy window of 250–750 keV (Fig. 4a). For the same energy window, the peak NECR of SimPET-XL was 349 kcps at 31.3 MBq, and the scatter fraction was 20% (Fig. 4b).

#### IQ

**Fig. 5:**
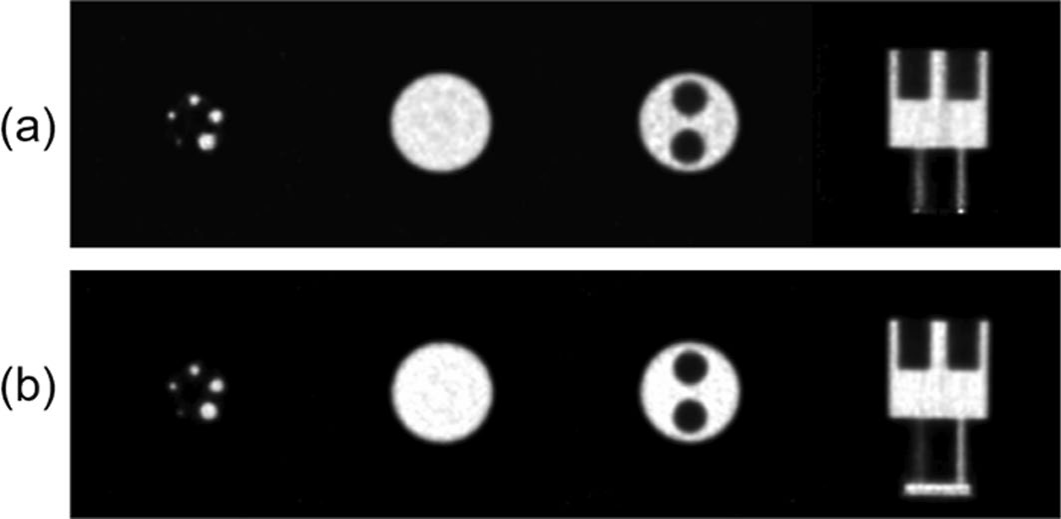
NEMA IQ phantom image with energy window of 250–750 keV for **a** SimPET-XL and **b** SimPET-L

Reconstructed NEMA IQ phantom images are shown in Fig. 5. SimPET-L provided a uniformity of the NEMA IQ phantom of 4.43% and SORs in air- and water-filled chambers of 5.54% and 4.10%, respectively. The RCs for 1, 2, 3, 4, and 5 mm rod diameters were 0.06, 0.55, 0.79, 0.84, and 0.90, respectively. SimPET-XL provided a uniformity of 3.89% and SORs in air- and water-filled chambers of 3.56% and 3.60%. The RCs for 1–5 mm rod diameters were 0.14, 0.62, 0.79, 0.91, and 0.95, respectively.

### Animal experiments

**Fig. 6:**
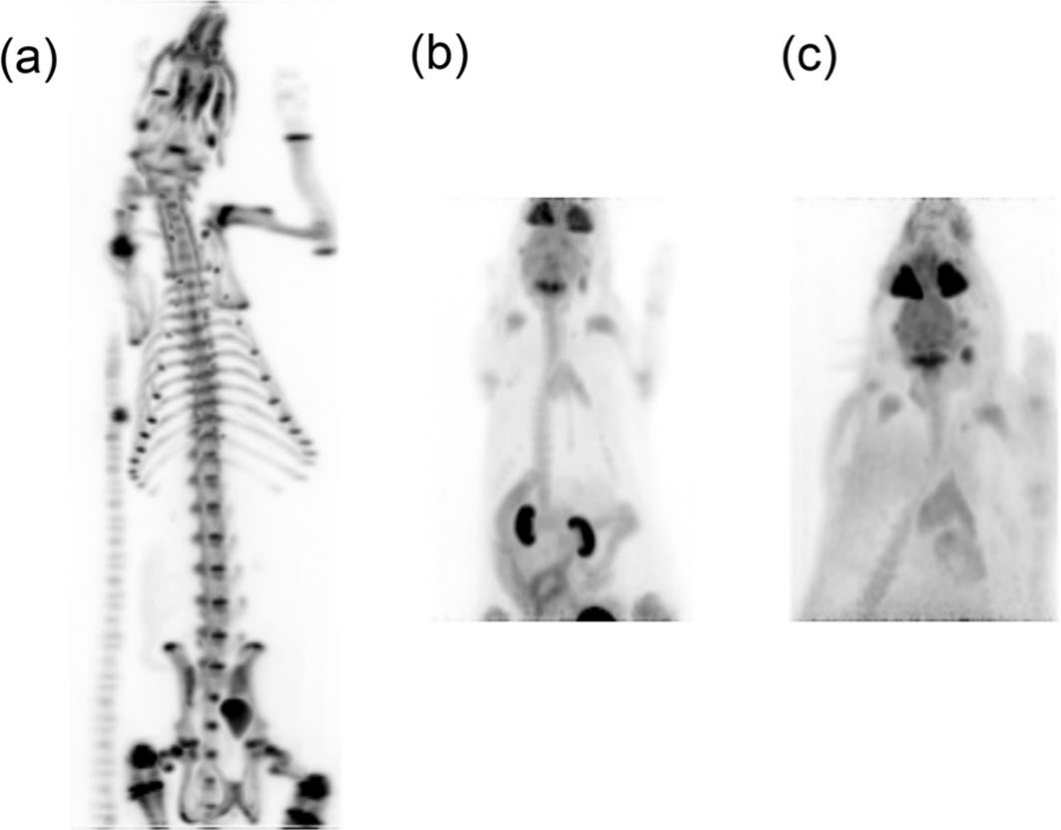
Acquired rat images. **a** ^18^F-NaF rat whole-body PET image, **b** ^18^F-FDG 6-week-old rat PET image, and **c** ^18^F-FDG 10-week-old rat PET image

The high sensitivity and resolution of SimPET-XL were demonstrated in maximum-intensity projection images of ^18^F-NaF, as shown in Fig. 6a. The detailed bone structures of the rats are clear. Figure 6b and c show images from a 20 min PET scan performed following tail vein injection of 33.6 and 33.4 MBq ^18^F-FDG.

## Discussion

The physical performance of SimPET-L and SimPET-XL was evaluated. These systems are PET inserts intended for imaging small animals in simultaneous PET/MRI with a 7.6 cm inner diameter. The evaluation was conducted in accordance with the NEMA NU4-2008 protocol and measurement methods in [18, 19]. To demonstrate the imaging capability of SimPET-XL with high resolution and sensitivity, PET imaging studies were performed on rats. The larger transaxial FOV of SimPET-XL and SimPET-L compared with preceding SimPET versions (SimPET™, SimPET-S, and SimPET-X) provided adequate space for imaging rats up to 500 g in weight when using the systems in standalone mode. In addition, SimPET-XL offered a relatively long axial FOV, increasing the sensitivity compared with SimPET-L (Tables 1 and 2).

**Table 1:** Device specifications of SimPET series

	SimPET-X	SimPET-L	SimPET-XL
Inner diameter (cm)	6.0	7.6	7.6
Outer diameter (cm)	9.9	11.2	11.2
Axial FOV (cm)	11	5.5	11

**Table 2:** Summary of PET performance

	SimPET-L	SimPET-XL
Radial resolution*		
Axial center	0.82 mm	0.91 mm
*Sensitivity*		
100–900 keV	6.30%	10.4%
250–750 keV	4.44%	7.25%
350–650 keV	3.31%	5.13%
400–600 keV	2.91%	4.46%
*Count-rate performance (250–750 keV)*		
Peak NECR	249 kcps	349 kcps
Activity at peak NECR	44.9 MBq	31.3 MBq
Scatter fraction	17.4%	20%
*IQ*		
Uniformity	4.43%	3.89%
RC at 1, 2, 3, 4, and 5 mm rod diameters	0.06, 0.55, 0.79, 0.84, and 0.90	0.14, 0.62, 0.79, 0.91, and 0.95
SOR air	5.54%	3.56%
SOR water	4.10%	3.60%

*IQ* image quality, *NECR* noise-equivalent count rate, *RC* recovery coefficient, *SOR* spill-over ratio

*Radial resolution measured using OSEM-PSF

As shown in Table 2, the sensitivities and peak NECRs of SimPET-XL were substantially higher than those of SimPET-L owing to the long axial FOV. In addition, the performance of SimPET-XL was not notably worse than that of SimPET-X, although SimPET-XL has a larger detector ring diameter than SimPET-X. The peak NECRs with energy window of 250–750 keV were similar, being 348 kcps at 26.2 MBq for SimPET-X and 349 kcps at 31.3 MBq for SimPET-XL. Although the scatter fraction of SimPET-XL was worse than that of SimPET-L owing to the long axial FOV, the scatter fractions of both systems were lower than those of SimPET™ and SimPET-X, which have smaller ring diameters [18].

Rat imaging studies were performed using SimPET-XL. The imaging capability of SimPET systems for mice with high IQ has been demonstrated in [18, 19]. Extending the axial length from 5.5 to 11 cm, inner diameter of the scanner from 6.0 to 7.6 cm, and detector face-to-face distance from 6.3 to 7.8 cm enabled PET imaging of the rat body. In addition, acquiring rat-body images in two sequential bed positions and stitching the reconstructed images allowed whole-body rat imaging (Fig. 6a).

In this study, we only measured the physical performance of the new SimPET series systems outside the MRI magnet. Although our preliminary studies on mutual interference between SimPET-XL and 9.4 T MRI did not show remarkable degradation of PET and MRI performances by combining the two modalities, more rigorous studies on the potential mutual interference are being conducted for verification.

## Conclusions

SimPET-L and SimPET-XL show comparable performance to the previous systems in the SimPET series. The larger transaxial FOV of these systems allows rat imaging studies with high IQ.

## Data Availability

All data generated or analyzed during this study are included in this published article.
